# CARRS Surveillance study: design and methods to assess burdens from multiple perspectives

**DOI:** 10.1186/1471-2458-12-701

**Published:** 2012-08-28

**Authors:** Manisha Nair, Mohammed K Ali, Vamadevan S Ajay, Roopa Shivashankar, Viswanathan Mohan, Rajendra Pradeepa, Mohan Deepa, Hassan M Khan, Muhammad M Kadir, Zafar A Fatmi, K Srinath Reddy, Nikhil Tandon, KM Venkat Narayan, Dorairaj Prabhakaran

**Affiliations:** 1 Public Health Foundation of India (PHFI), New Delhi & CoE-CARRS, PHFI, New Delhi, India; 2 Emory University, Atlanta, USA; 3 CoE-CARRS, PHFI, New Delhi, India; 4 CoE-CARRS, Public Health Foundation of India & Centre for Chronic Disease Control (CCDC), Tower No. 4, Commercial Complex, Sector-C, Pocket-9, Vasant Kunj, New Delhi, 110070, India; 5 Madras Diabetes Research Foundation (MDRF), Chennai, India; 6 MDRF, Chennai, India; 7 Aga Khan University, Karachi, Pakistan; 8 PHFI, New Delhi, India; 9 All India Institute of Medical Sciences (AIIMS), New Delhi, India; 10 Emory University, Atlanta, USA

**Keywords:** “Cardio-metabolic diseases”, Surveillance, Risk-factors, South-Asia

## Abstract

**Background:**

Cardio-metabolic diseases (CMDs) are a growing public health problem, but data on incidence, trends, and costs in developing countries is scarce. Comprehensive and standardised surveillance for non-communicable diseases was recommended at the United Nations High-level meeting in 2011.

Aims: To develop a model surveillance system for CMDs and risk factors that could be adopted for continued assessment of burdens from multiple perspectives in South-Asian countries.

**Methods:**

Design: Hybrid model with two cross-sectional serial surveys three years apart to monitor trend, with a three-year prospective follow-up of the first cohort.

Sites: Three urban settings (Chennai and New Delhi in India; Karachi in Pakistan), 4000 participants in each site stratified by gender and age.

Sampling methodology: Multi-stage cluster random sampling; followed by within-household participant selection through a combination of Health Information National Trends Study (HINTS) and Kish methods.

Culturally-appropriate and methodologically-relevant data collection instruments were developed to gather information on CMDs and their risk factors; quality of life, health-care utilisation and costs, along with objective measures of anthropometric, clinical and biochemical parameters. The cohort follow-up is designed as a pilot study to understand the feasibility of estimating incidence of risk factors, disease events, morbidity, and mortality.

**Results:**

The overall participant response rate in the first cross-sectional survey was 94.1% (Chennai 92.4%, n = 4943; Delhi 95.7%, n = 4425; Karachi 94.3%, n = 4016). 51.8% of the participants were females, 61.6% < 45years, 27.5% 45–60years and 10.9% >60 years.

**Discussion:**

This surveillance model will generate data on prevalence and trends; help study the complex life-course patterns of CMDs, and provide a platform for developing and testing interventions and tools for prevention and control of CMDs in South-Asia. It will also help understanding the challenges and opportunities in establishing a surveillance system across countries.

## Background

Cardio-metabolic diseases (CMDs) broadly comprise of diabetes mellitus, cardiovascular diseases (CVD), chronic kidney disease (CKD) and their common interconnected risk factors such as obesity, insulin resistance, glucose intolerance, dyslipidaemia, and hypertension. They are a growing public health problem worldwide [1] accompanying socioeconomic and nutrition transitions [2-4]. Coronary heart disease (CHD), cerebrovascular disease, and diabetes together account for 30% of global mortality and 80% of these deaths occur in low-and-middle-income countries (LMICs) [2,5-7]. In 2010, globally, 4,000,000 deaths were due to diabetes, the highest in absolute numbers was (1,008,000) in India [8]. The largest fraction of deaths from CHD (37%) and stroke (30%) attributable to high blood glucose were in South-Asia [9]. Further, in people of South-Asian origin, onset of diabetes [10-12], other cardio-metabolic risk factors [13,14], and late-stage disease events [15,16] occur at lower body mass indices and younger ages than other ethnic groups [16-23].

Key recommendations of the 2011 United Nations high-level meeting on non-communicable diseases (NCDs) and the US Institute of Medicine are initiation and strengthening of surveillance for NCDs [24] and the creation of integrated, comprehensive, sustainable, on-going nationwide surveillance systems [25]. In South-Asia, current efforts are limited to local surveys with vast state-wise heterogeneity and variable data quality [26-28]. Furthermore, projections of national income losses related to CMDs are based on models using inputs from limited local studies [29]; data on individual and household costs and social burdens are also scarce [30]. Current efforts by the Governments of India and Pakistan in setting up nationwide surveillance of NCDs are limited to self-reported surveys [31,32]. A robust surveillance system would need to be representative of the population of interest, utilise standardised methods that are not solely reliant on self-reporting, be amenable to scaling up, would be sustainably financed by the country/region itself, and also become a platform for further research opportunities and policy guidance (much like the role of the Centres for Disease Control and Prevention [CDC] in the United States) [33,34].

We present the design and methods of a model surveillance system for CMDs, the CARRS (**C**entre for c**A**rdiometabolic **R**isk **R**eduction in **S**outh-Asia)-Surveillance Study, which could be adopted for continuing assessments of burdens in South-Asian countries. The CARRS-Surveillance study builds on the WHO STEPS (World Health Organisation stepwise Approach to Surveillance) model [35] to capture prevalence of risk factors, CMDs, and their socioeconomic impact in serial representative surveys to understand trends, but goes a step further to convert the cross-sectional survey into a large, urban, sub-continent wide prospective cohort at lower-costs, to understand the incidence of risk factors, diseases, complications, and mortality. Thus, apart from estimating burdens, it can be used to develop South-Asian assessment and clinical management systems to tailor care and preventive approaches.

## Methods

### Study design

This is a hybrid cohort-modelled cross-sectional multi-centre surveillance study to be conducted over a period of four years. Two cross-sectional surveys conducted three years apart on standalone representative samples of each of the three city-wide populations, using objective measures will permit estimation of the prevalence and trends of CMDs and their risk factors. Those enrolled in the first cross-sectional survey will be followed as a cohort in a three-year study to estimate (i) the incidence of new risk factors (such as obesity, hypertension, diabetes,); (ii) incidence of later-stage target organ diseases such as peripheral vascular disease, stroke, myocardial infarction, congestive heart failure, chronic stable angina, CKD, retinopathy, neuropathy, and amputation; (iii) assessment of health service utilisation and costs including hospitalisation and outpatient use and (iv) morbidity and mortality associated with CMDs.

The first cross-sectional survey has been completed with ongoing first year of cohort-follow-up. The survey was comprehensive, undertaking assessments of quality of Life (QoL), and socioeconomic burdens on individuals and families with regards to these diseases. Participants underwent anthropometric measurements, blood pressure (BP) assessment, and provided biochemical specimens. The cohort follow-up was limited to patient reports with recording of BP and anthropometry. CMDs and their complications were diagnosed using standard definitions and coded using the International Classification of Diseases 10 (ICD-10) codes.

The study sites are metropolitan urban settings with large, growing (due to continued births and migration from various parts of the country), and heterogeneous populations. Estimates suggest that population size in Chennai (4.68 million) [36], Karachi (13 million) [37], and Delhi (16.3 million) [36], and the diversity in their composition make these cities current and future archetypes of rapid socio-economic, demographic, epidemiologic, and nutrition/lifestyle transitions in the South-Asian region.

### Sample size estimation

Utilising risk factor prevalence estimates from previously published Indian and Pakistani studies and anticipating a response rate of 80% with a design effect factor of 1.5 (to account for cluster sampling), the sample size estimates were generated for males and females in three age strata in each urban setting. As shown in table 1, the highest required sample size (3983 rounded-off to 4000 participants) permits each site to reliably estimate one or more of the CMD risk factors for each of the gender and age strata leading to a total sample size of 12,000.

**Table 1:** Sample size estimation (per site)

**Risk factors**	**Level of confidence**	**Margin of error**	**Baseline levels of indicators**	**Design effect**	**Expected response rate**	**No. of age/sex estimates**	**Sample size**
Tobacco use	1.96	0.05	0.23	1.5	0.8	6	3062
Hypertension	1.96	0.05	0.36	1.5	0.8	6	**3983**
Diabetes	1.96	0.05	0.15	1.5	0.8	6	2204
Overweight (BMI ≥ 23)	1.96	0.05	0.65	1.5	0.8	6	3933

With regards to the cohort follow-up, separate consent has been taken from participants to be followed up for three years or longer. An overall 15 - 25% loss-to-follow-up by the 3-year data collection time-period is anticipated due to the high probability of migration among the young population for job opportunities, marriage (in case of females), etc. Retention efforts (in the form of maintaining updated contact information; collecting contact details of friends and relatives; periodic reminder calls; courtesy calls/visits) have been put in place to keep track of participants and minimise loss-to-follow-up. Although the study at present is not powered to estimate incidence of CMDs and their risk factors, it has the potential to determine such incidence rates if the follow-up period is increased and the study is scaled up by adding follow up of subsequent cross-sectional samples.

### Sampling method

Households were selected in each of the three cities using a multi-stage cluster random sampling technique. Each city has its own distinctive municipal sub-divisions, encompassing municipal corporations, wards and Census Enumeration Blocks (CEB), which were used sequentially as sampling frames to randomly select households. While wards were the primary sampling units (PSUs) for Chennai and Delhi, CEBs or clusters were the PSUs for Karachi. STATA version 10.1 (Statacorp, TX) [38] and data from the most recent census were used to randomly select the wards, CEBs, and households (defined below.). To give each household an equal chance of being selected for the study and to identify households constructed after the last census survey, manual listing and mapping of all households in each CEB was done before randomly selecting them.

Two participants, one male and one female, aged 20 years or older, were selected from each household based on inclusion and exclusion criteria given below. Two methods were used for within household sampling – (i) for households with one to two adults (≥20 years), the sampling strategy described in the 2002 Health Information National Trends Study (HINTS) in the USA was used [39]. According to HINTS, one or both individuals (one male and one female) were selected and enrolled into the study based on eligibility criteria and informed consent; (ii) for households with more than two eligible adults, the “Kish method” used in the WHO’s STEPS surveys [35] was applied. Recruitment of participants, and data and specimen collection were conducted through three visits to each participant’s place of residence, respectively (Visit-0, Visit-1, and Visit-2).

### Inclusion and exclusion criteria for CARRS – Surveillance Study

#### Inclusion criteria

Any individual aged ≥20 years and permanently residing in the selected household.

For the purpose of this study, a permanent resident was defined as a person living in the selected household, was related to the household head and ate at least 3 meals in a week with the family.

*Households were defined as “a group of people who live together, usually pool their income and eat at least one meal together a day when they are at home. This does not include people who have migrated permanently or are considered visitors” [(Integrated Disease Surveillance Project (IDSP)]*[31]*.*

#### Exclusion criteria

Pregnant women were not included in the study since their biochemical parameters would vary from the normal physiology due to pregnancy, further their patterns of diet and physical activity would also be different from usual.

Bed-ridden individuals were excluded because of the difficulty in taking anthropometric measurements in these individuals. However, reasons for being confined to bed were collected from such individuals to estimate prevalence of CMDs among this excluded group (since CMDs can be the cause for being bed-ridden).

### Surveillance indicators and study instruments

To provide consistency and reproducibility of the results across multiple sites, comprehensive and uniform data collection instruments were used to capture measurements (table 2). Household data were collected through interviewer administered paper questionnaires in English or the preferred local languages (Hindi, Tamil, and Urdu). Validated questions were derived from English questionnaires used in the WHO Multinational MONItoring of trends and determinants in CArdiovascular disease (MONICA) study [40], WHO STEPS studies [35], and from previous regional and national surveys. Using these, culturally-appropriate and methodologically-relevant closed-questions, an instrument for South-Asia was developed and pilot tested for face and construct validity prior to use in the study. Several sections of the baseline questionnaire (such as the QoL, CMD history, tobacco and alcohol consumption questions) were based on validated questionnaires that already exist in regional languages (Tamil, Hindi and Urdu). Questionnaires to elicit medical and treatment history, costs and QoL are being used to collect incident events during the ongoing cohort follow-up. Further, verbal autopsy is being performed using a reliable instrument to ascertain cause of death of participants who die during the course of follow-up and for whom either death certificate was unavailable or cause of death not certified. For the adapted instruments collecting a variety of CMD risks and diseases (e.g. tobacco, history of CMD, heart failure, and Chronic Obstructive Pulmonary Disease), the subjective history provided by participants was validated against laboratory and other diagnostic gold standards (e.g., salivary cotinine for tobacco consumption). These in-built steps to validate the self-reported data distinguishes the CARRS-Surveillance as a stronger model compared to the IDSP [31] and the INDEPTH network (International Network of field sites for continuous Demographic Evaluation of Populations and Their Health in developing countries ( http://www.indepth-network.org) [41]. 

**Table 2:** Summary of the surveillance indicators, measures, methods and instruments

**Indicators**	**Measures**	**Methods**	**Instruments**
Demographic and Social Characteristics*	Age / Sex / Marital Status / Religion	Questionnaires	Chennai Urban Population Study (CUPS), Chennai Urban Rural Epidemiological Study (CURES), Establishment of Sentinel Surveillance System for CVD in Indian Industrial Populations (Sentinel Surveillance Study)
	Education / Income / Occupation		
	Household assets		Standard of Living Index (SLI)
	Contact Details (and supplemental contacts)		
Behavioral risk factors*	Tobacco use	Questionnaire / Cotinine in saliva (5 % of participants)	CUPS, CURES, Sentinel Surveillance Study
	Alcohol use	Questionnaire	
	Dietary habits	Questionnaire/ validation by 24-hour dietary recall in a sub-sample	INTERHEART Study
	Physical activity	Questionnaire	International Physical Activity Questionnaire (IPAQ)– short
	Sleep		Sleep Heart Health Study (SHHS)
Physiological and biochemical risk factors**	Hypertension	Blood pressure measurement	Standardized method (American Heart Association) and validated instrument (certified by British Hypertensive Society and Association for the Advancement of Medical Instrumentation)
	Dyslipidemia	Laboratory estimation of serum total cholesterol, low density lipoprotein cholesterol, very low density lipoprotein cholesterol, high density lipoprotein cholesterol, triglycerides, Apolipoprotein A and B (not done in Karachi)	Standardized across all three study sites
	Obesity	Anthropometry (height / weight / body circumferences / skinfold thickness / body composition/bio-impedance)	Standard procedures based on National Health And Nutrition Examination Survey-III with instruments used in epidemiological studies on South Asian population
	Diabetes	Laboratory estimation of fasting plasma glucose, glycated haemoglobin (HbA_1_c)	Standardized across all three study sites
Female Reproductive history*	Menarche/ gestational history (pregnancy induced hypertension, gestational diabetes), menopause (surgical / physiological / whether on hormone replacement therapy) / contraception	Questionnaire	CUPS, CURES, India Health Study (IHS)
Quality of Life*	Mobility, self care, usual activities, pain/discomfort, anxiety/depression (related to cardiometabolic diseases; CMDs and their risk factors)	Questionnaire	European Quality of Life 5 Dimensions questionnaire (EQ-5D)
Morbidity**	Stroke / Myocardial infarction / Congestive heart failure / Chronic stable angina	Questionnaires including medication history;	Rose Angina, CURES, IHS, Sentinel Surveillance, Community Heart Failure questionnaire
		Medical records of documented events or procedures, serum urea and creatinine and albumin for CKD	
	Chronic kidney disease (CKD)/ Dialysis / Renal transplantation		
	Amputation/diabetes retinopathy		
	Procedures, Revascularization, Hospitalization		Initiative for Cardiovascular Health Research in the developing countries (IC-Health) macroeconomic study
Treatment history, health services, quality of care and health care costs**	Awareness and risk factor control	Questionnaire	IC-Health macroeconomic study
	Access to health care services		
	Utilization of services		
	Health insurance / coverage		
	Costs of treating CMDs and their risk factors		
Chronic Obstructive Pulmonary Disease (COPD), Asthma*	Prevalence of COPD & asthma in the population	Questionnaire	NHANES III and the present standards of the American Thoracic Society (ATS)
Family history*	Prevalence of CMDs and their risk factors in members of the family related to the participants	Questionnaire	Action to Control Cardiovascular Risk in Diabetes (ACCORD) trial
Mortality***	All cause	Follow-up surveys; Death Certificates; Verbal Autopsy	Modified version of Registrar General of India – Center for Global Health Research (RGI-CGHR) Prospective Study on Million Deaths (Form 10C)
	Cardiovascular disease specific; Diabetes-specific		

*Only cross-sectional surveys; **both cross-sectional survey and cohort follow-up; ***only cohort follow-up.

### Biological sample collection and storage

Biological sample collection involved drawing 15 ml of blood (in fasting state) from each participant, collecting urine (early morning void), and 1000 to 2000 μl of saliva (fasting) in Salivettes. While blood and urine were collected from all participants, saliva was collected from 5% of the study participants (i.e. 200 participants per site). The samples were transported from field sites in cold chain to the laboratories for analysis. Sample aliquots were also stored in cryo-vials at - 80 degrees Celsius for future studies. The methods of analysis and external quality control have been standardised for all biological samples across the study sites (table 3). There is one exception in that Apolipoprotein A and B analyses were not conducted in Karachi due to lack of required laboratory facilities for the test.

**Table 3:** Biological samples and their methods of analysis

**Clinical parameter**	**Laboratory parameter**	**Methods used for analysis**		
		**Chennai**	**Delhi**	**Karachi**
Diabetes	Fasting plasma glucose	Hexokinase/Kinetic	Hexokinase/Kinetic	Glucose Oxidase / End Point
	Glycated haemoglobin (HbA1c)	High performance liquid chromatography (HPLC)	HPLC	HPLC
Dyslipidemia	Total cholesterol	Cholesterol Oxidase Peroxidase (CHOD-POD) end point	CHOD-POD end point	Enzymatic Colorimetric method (CHOD-PAP)
	High density lipoprotein cholesterol	Direct	Direct	Direct
	Low density lipoprotein cholesterol	Friedwald Formula	Friedwald Formula	Friedwald Formula
	Very low density lipoprotein cholesterol	Calculation	Calculation	Calculation
	Triglycerides	Enzymatic methods (GPO-PAP end point)	Enzymatic methods (GPO-PAP end point)	Enzymatic methods (GPO-PAP end point)
	Apolipoprotein A, Apolipoprotein B	Immuno-turbidimetric	Immuno-turbidimetric	Will not be done
Kidney disease	Serum urea	Urease Glutamate Dehydrogenase (GLDH) / Kinetic	Urease GLDH/ Kinetic	Blood Urea Nitrogen (BUN): Enzymatic conductivity rate method
	Serum creatinine	Jaffe Kinetic	Jaffe Kinetic	Modified Jaffe’s Method
	Microalbuminuria	Immuno-turbidimetry	Immuno-turbidimetry	Rate nephelometry
Tobacco exposure	Salivary cotinine	Elisa kit	Elisa kit	Elisa kit

### Clinical and anthropometric assessments

Two clinical (BP and pulse rate) and eight anthropometric measurements of participants were taken during the visits: Clinical measurements - BP and Pulse rate. Anthropometric measurements - Mid-arm circumference, Waist circumference, Hip circumference, Triceps skin-fold, Sub-scapular skin-fold, Supra-patellar skin-fold, Height (Standing) and Body composition analysis by Bio-impedance.

The equipment and methods used for BP and anthropometric measurements were standardised and certified, and have been used in other epidemiological studies in the South-Asian population. BP was measured using electronic sphygmomanometer; Omron HEM-7080 and HEM-7080IT-E; Omron Corporation, Tokyo, Japan (certified by the British Hypertensive Society and the American association for Advancement of Medical Instrumentation [AAMI] protocols). Skinfold Calipers (Holtain Ltd., UK) and non-stretch measuring tape (Gulick II, Country Technology, Gays Mills, WI) were used to measure skin-fold thickness and body circumferences, respectively. Height was measured using a portable Stadiometer (SECA Model 213, SecaGmbh Co, Hamburg, Germany). Apart from these, body-composition analysers (instrument which measures body fat by sending out weak electric currents to measure impedance/electrical resistance by different tissues of the body); Tanita BC-418 in Delhi and Chennai, and BC-545 in Karachi were used to measure compartmental body fat distribution. To ensure standardisation, both instruments were tested in 50 male and 50 female participants to compare the parameters measured; i.e. weight, body mass index, basal metabolic rate, body fat and visceral fat. The results showed that all measured parameters were highly co-related for both males and females (r > 0.95, p < 0.05) between the two instruments, except body fat in males (r = 0.67, p = 0.67). Methods for BP measurement and anthropometric measures were based on the recommendation of the American Heart Association’s Council on High Blood Pressure Research [42] and the third National Health and Nutrition Examination Survey (NHANES-III) [43].

### Data management

An online system was developed in an ‘open source’ platform PHP (Hypertext PreProcessor, scripting language for the web page/front end) and MySQL (My Structured Query Language) for data entry and database management at each site. This online database has been programmed to have automated in-built checks for logic which are ‘clinically reasonable’ (such as ranges, absolute and relative values, context and structure). It provides an efficient means of data entry, storage, and quality control. Data are available at the coordinating site for immediate feedback and timely corrections. The data have been stored in pass-word protected files and questionnaires in locked cabinets in all study sites, and only the study personnel have access to these. All information related to participant identification was de-linked from the data files before analysis to maintain anonymity.

### Quality control strategies

Quality control (QC) strategies were applied using a framework which comprehensively considers each phase of the study and applies inter-related themes to every level of the study and are described in table 4. Apart from standardisation of laboratory methods (table 3), QC involved laboratory procedure assessment at two levels. Level-1, internal quality control: Local laboratories attached with the study centre followed their own internal quality control standard operating procedures (SOPs) to ensure accuracy, precision, and reproducibility. Level-2; external quality control: Irrespective of the nature of existing laboratory accreditation and / or SOP’s, all study site laboratories were enrolled into an external quality assessment program for clinical chemistry, HbA1c (glycated haemoglobin), lipid and human urine. This was implemented with support from the Randox International Quality Assurance Scheme (RIQAS), UK. The frequency of external quality control sample was two per month for clinical chemistry, lipid and urine, and one per month for HbA1c.

**Table 4:** Quality assurance strategies

**Levels of quality control**	**Phases**			
	**Design and planning**	**Pilot testing**	**Data collection**	**Data analysis**
	● Critical review of protocols	● Fluidity and feasibility of field operations assessed	● Monitoring field activities	● Audit and evaluate validity of findings prior to publication
**Coordinating center**				
	● Common manual of operations for three study sites			
				● Internal peer reviews prior to publication
	● Coordination of timelines & activities			
**Investigators**	● Reviewed the design and planning of the study	● Results were audited after completion of the pilot	● Monitoring	● Validity checks
				● Results reviewed
	● Regular steering committee meetings			
**Field Personnel**	● Extensive training over a period of 7–10 days – theory and practical, field visits and shadowing by the study managers	● Evaluated all field and documenting techniques	● Random checks, re-training	
	● Easy-to-carry operations guide provided			
**Survey Questionnaires**	● Peer-reviewed	● Established clarity and face validity in small field sample	● Regular checks done to assess completeness	● Compromised or inadequately completed questionnaires identify and discard
	● Translated into local languages			
	● Internal consistency estimates and reliability exercises through review of literature on survey instruments and their published data			
**Measuring Equipment**	● Centrally procured	● Evaluated calibration techniques, acceptability of use in field	● Regular calibration of equipment; faulty equipment replaced as and when required	
	● Central training			
	● Calibration guidelines and checks developed			
**Specimens**	● Kits and equipment procured centrally	● Evaluated adherence to protocols, labeling, processing, storage and handling ● Interim analysis conducted to detect outliers	● Random checks done	● Samples stored for future investigation
			● External temperature gauge labels to monitor sample temperature	
				● Compromised samples identify and discard
	● Specific protocols for each biochemical assay was developed			
	● Extensive training (labeling, handling, storage)			
**Laboratory**	● Laboratory selected and reference laboratory identified based on National Accreditation Board for Testing and Calibration Laboratories, Department of Science and Technology, Government of India (NABL) or College of American Pathologists, Northfield, IL, USA(CAP) certification	● Evaluated procedural fluidity	● Internal quality checks and calibration	Assessment of intra- and inter-laboratory coefficients of variation
			●Regular external validation – lyophilized samples from reference laboratory	
		● Evaluated intra- and inter-laboratory variability		
		● Analysis conducted to detect outliers		
	● Internal and external quality assessment protocols and schedule of regularity developed			
**Communication**	● Reporting structures were established	● Agility of transfers assessed		
	● Data transfer planned			
**Documentation**	● Checklists and logbooks were maintained	● Recording legibility assessed		● Audit logbooks for response rates and field activity indicators maintained
	Training in appropriate and legible documentation			
**Data Storage & Confidentiality**	● Data back-up and protection policies have been established	● Accessibility, simplicity and flexibility of software assessed	● Locked and password-protected data storage	● Datasets de-identified
				● Access to personal identifiers limited
			● Active back-up	
	● Training of all staff			
**Data Entry**	● Protocols, consistent data cleaning methods and verification systems were established	● Variability assessments conducted	● Interim analyses to identify duplicate entries	● Reporting on outliers
				● Validity checks
			● Decision log to document issues	
				● Database errors tracked

## Results

The first cross-sectional study was conducted between September 2010 and November 2011. A total of 14, 215 individuals in 8, 115 households were approached in the three study sites (5348 participants in Chennai, 4609 in Delhi, and 4258 in Karachi). From these, a total of 13,384 participants were recruited (4943 Chennai, 4425 Delhi, and 4016 Karachi). A total of 831 eligible participants in the three sites refused to participate in the study; overall response rate thus was 94.1% at the participant level (Chennai 92.4%, Delhi 95.7%, Karachi 94.3%). About eighty-one percent of the participants recruited into the study contributed biospecimens (Chennai 83.9%, Delhi 79.8% and Karachi 87.2%). Overall, 51.8% of the participants were females; a large proportion of the sample, 61.6% was below the age of 45 years, 27.5% in the 45–60 years age group and 10.9% > 60 years of age. In addition, the study provides an understanding of the existing political and social challenges in establishing a sustainable surveillance system for CMDs in the two countries and evidences of context specific successful measures that can be adopted to overcome most of these challenges (summarised in table 5).

**Table 5:** Challenges in the implementation of the study and methods used to overcome them

	**Challenges**	**Solutions**
**Mapping and listing of households**	Reference data Delhi and Chennai: 2001 census. Karachi: 1998 census.	Complete listing of all the households in all randomly selected CEBs was done by field workers and structural maps of the areas were developed manually.
	Lot of changes in structure and population had taken place by 2010	
**Training of trainers (ToT) and site managers for uniform implementation of the study**	Challenges with regards to organising the ToT in either India or Pakistan due to visa issues for trainers and participants.	The ToT was organised in Kathmandu, Nepal with assistance from the Nepal Public Health Foundation.
**Participant recruitment and interviews**	Poor response from upper socioeconomic status localities and gated communities	Resident Welfare Association, Societies and Unions of the localities were approached for cooperation.
	Recruiting and interviewing male participants - who could not be contacted on working days	Interviews were scheduled on weekends, early mornings and late evenings, and more field workers were recruited to conduct these weekend surveys.
	Frequent electricity breakdowns in Karachi in the evenings.	Emergency lights were arranged for interviewing the participants in the evenings.
	The socio-political climate in Karachi posed challenges to the safety of interviewers and in completion of surveys.	Field work was scheduled accordingly to target safe areas as per the socio-political situation of the city on a day-to-day basis.
**Blood sample collection**	Fear of providing blood samples among the participants of lower socio economic status	Team leader and supervisor contacted and counselled the participants.
	Not coming fasting to the blood collection camps – some participants consumed tea or juices early in the morning.	The blood samples for these participants were not collected in the camp on that day, but were collected on another day from their homes ensuring that the participant was in fasting state. The samples were transported to the laboratory in appropriate cold chain.
	Blood samples could not be collected during the month of Ramadan (Islamic fasting month) in Karachi.	During the month of Ramadan, non-Muslim participants (mainly from the Christian communities) were recruited.
	Difficulty in conducting blood collection camps during extreme (cold and hot) weather conditions.	As far as possible camps were avoided on extreme cold and hot days in Delhi.
**Anthropometry**	The instrument purchased for the other two sites Tanita BC-418 was not available at Karachi and also could not be shipped in to the country.	A different model of Tanita was used in Karachi, BC-554, but the two models were compared by measuring the correlation of their parameters in 100 participants (described in the text).
Bio-impedance		

## Discussion

The CARRS-Surveillance model has been established in Chennai and New Delhi in India, and Karachi in Pakistan with successful completion of the first cross-sectional survey and initiation of the first year of cohort follow-up. The response rate for the first cross-sectional survey was more than 90% in all study sites with an overall rate of 94%. The combined cross-sectional and longitudinal study design lends it the advantages of:

(i) serial cross-sectional survey based models such as the NHANES [43] and the Behaviour Risk Factor Surveillance Surveys (BRFSS) in the USA [44] and Jordan [45], and the national NCD risk factor examination surveys in Seychelles [46] and Cuba [47];

(ii) longitudinal prospective models such as the SCORE (Systematic COronary Risk Evaluation) project which helped to develop a risk scoring system for management of CVD in Europe [48] and other longitudinal studies which helped to estimate the psychosocial risk factors of CHD [49].

CMDs are among the top ten most costly diseases [50], but have the advantage of being largely predictable through identification of distal and intermediate risk factors, and also substantially preventable through changes in lifestyle and/or use of preventive pharmacology [1,51,52]. The surveillance model if scaled up has the potential to estimate secular trends and incidence rates of mortality and morbidity due to CMDs, their complications and risk factors, thereby providing means of prioritizing and measuring the impact of public health interventions.

In a recent review of prevailing methods of NCD surveillance, particularly of CVD in India, the authors reiterate the need for harmonising all existing efforts, at least in measurement tools and quality assurance methods, to establish an integrated, comprehensive, and standardised surveillance system [53]. CARRS-Surveillance provides an understanding of the challenges in establishing such a surveillance system for CMDs, and elucidates the means to address them. However, we suggest that such an effort should not be limited to individual countries, but should be consolidated for South-Asia as a whole because the entire region is experiencing an epidemiological transition leading to increased incidence of CMDs and their risk factors. Also, there are shared demographic, socio-economic, behavioural, and physiological determinants among South-Asians. One such multi-site collaborating surveillance network is INDEPTH which regularly collates cross-sectional survey data from 34 sites in 17 LMICs [41]. However, these are based on the self-selected samples of Health and Demographic Surveillance Systems (HDSS) in each site and are only representative of a district, therefore, the findings cannot be generalised to the region or the country [41].

### Strengths and limitations

Apart from robust study methods and quality control mechanisms, the sample population recruited in our study conforms to the current age profile of the population in the two countries; about 65% of the population in India and 75% of population in Pakistan are below the age of 35 years [36,54]. This demonstrates the success of the sampling strategy employed and has implications for the generalisability of findings. However, a limitation of the CARRS model is that the study setting is urban and does not include the larger rural population where the burden of CMD is also growing. An urban model, however, would anticipate the growth of urban areas the world over, and also provide insights into operational aspects of surveillance systems, and empirical evidence of successful implementation at lower costs. The New York City Health and Nutrition Examination Survey (NYC HANES) provides an aspirational model suggesting that surveillance in such metropolitan cities with diverse populations might provide a reasonable reflection of diverse and growing cities to each individual nation’s disease burdens [55].

Several LMICs have some structure to estimate the burden of NCDs, but a recent study by the WHO in 23 high burden countries (which includes India) showed that the existing systems are deficient in standardised data collection tools and often lack accuracy and quality [56]. The CARRS-Surveillance model addresses these technical standardisation and quality challenges in setting up national and regional CMD surveillance systems in South-Asia, but the task of scaling up will require political commitment, funds, and human resources. Although challenging, this is achievable and has been accomplished by a few LMICs. For example, the Ministry of Health in Jordan, in partnership with WHO and CDC, established the Jordan BRFSS in 2002 which conducts cross-sectional surveys every two to three years [45]. The national examination survey of NCD risk factors in Seychelles has been collecting data for planning and evaluating interventions since 1989 [46], and the Cuban system since 1995–96 [47]. Eleven Latin American countries (Argentina, Brazil, Chile, Colombia, Dominican Republic, Guatemala, Mexico, Panama, Peru, Uruguay and Venezuela) have new or emerging systems for serial national NCD and risk factor surveys in various stages of development [57]. These countries have demonstrated the utility of continuous surveillance in identifying high risk communities, planning interventions, and evaluating the effects of existing policies, thereby creating an evidence-base for steering national policies on NCD prevention and health promotion [45-47,57].

## Abbreviations

AAMI: American Association for Advancement of Medical Instrumentation; BMI: Body Mass Index; BP: Blood pressure; BRFSS: Behaviour Risk Factor Surveillance Surveys; CARRS: Centre for Cardiometabolic Risk Reduction in South-Asia; CDC: Centres for Disease Control and Prevention; CEB: Census enumeration blocks; CHD: Coronary heart disease; CKD: Chronic kidney disease; CMDs: Cardio-metabolic diseases; CVD: Cardiovascular diseases; DM: Diabetes mellitus; HbA1c: Glycated haemoglobin; HDSS: Health and Demographic Surveillance Systems; HINTS: Health Information National Trends Study; ICD-10: International Classification of Diseases 10 codes; IDSP: Integrated Disease Surveillance Project; LMICs: Low-and-middle-income countries; MONICA: Multinational MONItoring of trends and determinants in CArdiovascular disease; MySQL: My structured query language; NCDs: Non-communicable diseases; NHANES-III: National Health and Nutrition Examination Survey - third; NYC HANES: New York City Health and Nutrition Examination Survey; PHP: Hypertext preprocessor, scripting language for the web page/front end; PSUs: Primary sampling units; QC: Quality control; QoL: Quality of Life; RIQAS: Randox International Quality Assurance Scheme; SCORE: Systematic COronary Risk Evaluation; SOPs: Standard operating procedures; UK: United Kingdom; USA: United States of America; WHO: World Health Organisation; WHO STEP: World Health Organisation STEPwise approach to surveillance.

## Competing interests

The authors declare that there are no competing interests financial or non-financial with regards to this study. The interpretation of data and presentation of information is not influenced by any personal or financial relationship with any individual or organization.

## Authors’ contributions

All authors listed in the paper have contributed sufficiently to fulfil the criteria for authorship. Apart from this there is no other individual who has contributed sufficiently and who fulfil the criteria for authorship but has not been included as an author for this paper. All authors read and approved the final manuscript.

## COE-CARRS Surveillance Investigators’ Group

Steering Committee: Dorairaj Prabhakaran, K. M. Venkat Narayan, K Srinath Reddy, Nikhil Tandon, V. Mohan, Muhammed M. Kadir, Mohammed K. Ali, Vamadevan S Ajay

Operations: Dorairaj Prabhakaran, Nikhil Tandon, K. M. Venkat Narayan, Mohammed K Ali, Muhammed M. Kadir, S. Roopa, Hassan M. Khan, R. Pradeepa, M. Deepa, Vamadevan S Ajay, Dimple Kondal, Ruby Gupta, Pragya Sharma

Coordinating Centre (Delhi): Dorairaj Prabhakaran, Nikhil Tandon, S. Roopa, Vamadevan S Ajay, Manisha Nair, Nivedita Devasenapathy, Divya Pillai

Development of questionnaires and manual of operations: Dorairaj Prabhakaran, Nikhil Tandon, K. M. Venkat Nararayan, Mohammed K. Ali, Manisha Nair, Nivedita Devasenapathy, R. Pradeepa , Ed Gregg, Anwar Merchant, Romaina Iqbal

Data management and statistical team: Dimple Kondal, Shivam Pandey, Praggya, Naveen

Laboratory: Lakshmy Ramakrishnan, Ruby Gupta, Savita

Information Technology: Ramanathan K, Ansel J D’Cruz, Gnanashekaran K.

Online data entry software: Mahesh Dorairaj

Data collection team

Chennai:

Field supervisor: Rahul T

Field interviewers: Alagarsamy, Anthony JV, Arul Dass.A, Arul Pitchai.S, Ashok Kumar, Balaji V, Dhanasekar L, KalaiVani D, Kumar M, Nandhakumar, Prathiban K, Sampath, Saravana Kumar P, SaravananR, Senthil RajaR, ShenbagaValliE, SivamanikandanK, SureshT, Uma Sankari G

Laboratory assistants: Geetha Priya L, Gowri, Irin Jayakumari A, Padmapriya, Ramakrishnan R, Revathy, Satish Raj S, Sudha M, Suresh, Vijay Baskar S

Data entry operators: Narayanan, Nirmala

Delhi:

Field supervisor: Liladhar Dorlikar

Field interviewers: Parag Jyoti Das, Kulwant Kaur, Sweta Kumari, Meena Thakur, Garima Rautela, Avijeet Malik, Anita Yadav, Makhan, Rishi Garg, Arun

Laboratory assistants: Priyanka Nautiyal, Sunil Dogra, Geetha

Data entry operators: Naveen Kaushik, Avnish

Karachi:

Field supervisor: Mehboob John Samuel

Field interviewers & laboratory assistants: Yousuf Sadiq, Shukrat Khan, Shahirah Ziarat Khan, Nadia Khan, Noureen Khan, Naseem Sehar, Asif Shabaz, Fakhrah Perveen, Karan Inayat, Tajir Hussain, Tariq Hussain, Nasreen Khan

Data entry operator: Sayed Arif Hussain Kazmi

## Ethics approval

CARRS-surveillance study has been approved by the Institutional Review Boards (IRBs) of Public Health Foundation of India, New Delhi, All India Institute of Medical Sciences, New Delhi, Madras Diabetes Research Foundation, Chennai, India, Aga Khan University, Karachi, Pakistan, and Emory University, Atlanta, USA. In addition the study has received regulatory approval from the National Heart, Lung, and Blood Institute (NHLBI), National Institutes of Health (NIH), USA and the Health Ministry Screening Committee of India, New Delhi.

## Pre-publication history

The pre-publication history for this paper can be accessed here:

http://www.biomedcentral.com/1471-2458/12/701/prepub
